# Practical Chemistry

**Published:** 1852-01

**Authors:** 


### PRACTICAL CHEMISTRY.

From the following circular, it will be seen that an opportunity is offered to the young men in the West to acquire a practical knowledge of analytical chemistry, and we should hope there are not a few who will be inclined to profit by the excellent opportunity. There is a demand in our country for much more of that sort of knowledge than prevails, and those who make themselves thorough, practical chemists are pretty sure of finding profitable employment:

*Laboratory of Practical Chemistry, under the direction of Professor B. Silliman, Jr., A. M., M. D., assisted by Mr. Geo. J. Brush.*

Pupils are received in this Laboratory for instruction in Practical and Analytical Chemistry, assay of Ores and analysis of Minerals, Mineral Waters, products of the Arts and kindred subjects. The instruction in this Laboratory is entirely unconnected with the other operations of the University, but the pupils have the opportunity of following the Chemical Lectures given in the regular organization of the Medical Department. Students are furnished the use of glass, reagents and when sufficiently advanced with balances and other delicate means of research.—They can work during the whole day under the direction of the Assistant, whose whole time is entirely devoted to their progress and improvement. Daily instruction will also be given in some departments of Analytical and General Chemistry and in Mineralogy.

Cabinets of Minerals, Fossils, and other objects in Natural History will also be accessible.

This Laboratory is open from the first of October in each year until June.

Analysis of Ores, Minerals, Mineral Waters, products of the Arts, and cases of suspected poisoning will be executed with promptness and on reasonable terms.

For further information apply, by letter, to

PROF. B. SILLIMAN, JR., Louisville, Ky.

